# Supplementary material for: Incidence and persistence of asymptomatic Leishmania infection among HIV-infected patients in Trang province, Southern Thailand: A cohort study
Source: PLoS Negl Trop Dis. 2024 Oct 8;18(10):e0012581. doi: 10.1371/journal.pntd.0012581 (PMC11488742; doi:10.1371/journal.pntd.0012581)
Supplement: S1 Table — (DOCX) [file pntd.0012581.s001.docx]

**S1 Table. Characteristics of the study population by *Leishmania* infection status at baseline (2015-2016) and follow-up visit status (2018-2019)**

|  | | **Follow-up status among**  **negative *Leishmania* infection at baseline, n=479** | | | | | | | | **Follow-up status among**  **positive L*eishmania* infection at baseline, n=164** | | | | | | | |
| --- | --- | --- | --- | --- | --- | --- | --- | --- | --- | --- | --- | --- | --- | --- | --- | --- | --- |
| **Characteristics** | | **Yes**  **n=373 (%)** | | **No**  **n=106 (%)** | | **Total**  **n = 479 (%)** | | **P-value** | | **Yes**  **n=133 (%)** | | **No**  **n=31 (%)** | | **Total**  **n=164 (%)** | | **P-value** | |
| **Age** | |  | |  | |  | | 0.563 | |  | |  | |  | | 0.697 | |
| Mean (SD) | | 44.1 (8.2) | | 42.4 (9.1) | | 43.7 (8.4) | |  | | 43.1 (8.9) | | 42.7 (7.5) | | 43.1 (8.6) | |  | |
| Min-Max | | 21.6-71.3 | | 20.1-72.1 | | 20.1-72.1 | |  | | 20.1-73.7 | | 27.6-59.1 | | 20.1-73.7 | |  | |
| **Sex** | |  | |  | |  | | 0.211 | |  | |  | |  | | 0.165 | |
| Male | | 189 (50.7) | | 61 (57.5) | | 250 (52.2) | |  | | 62 (46.6) | | 19 (61.3) | | 81 (49.4) | |  | |
| Female | | 184 (49.3) | | 45 (42.5) | | 229 (47.8) | |  | | 71 (53.4) | | 12 (38.7) | | 83 (50.6) | |  | |
| **Education** | |  | |  | |  | | 0.134 | |  | |  | |  | | 0.606 | |
| Primary school and lower | | 162 (43.4) | | 46 (43.4) | | 208 (43.4) | |  | | 56 (42.1) | | 13 (41.9) | | 69 (42.1) | |  | |
| Secondary school | | 112 (30.0) | | 42 (39.6) | | 154 (32.1) | |  | | 34 (25.6) | | 11 (35.5) | | 45 (27.4) | |  | |
| Vocational school | | 46 (12.3) | | 7 (6.6) | | 53 (11.1) | |  | | 24 (18.0) | | 3 (9.7) | | 27 (16.5) | |  | |
| Bachelor or higher | | 53 (14.2) | | 11 (10.4) | | 64 (13.4) | |  | | 19 (14.3) | | 4 (12.9) | | 23 (14.0) | |  | |
| **Occupation** | |  | |  | |  | | 0.477 | |  | |  | |  | | 0.957 | |
| Unemployed | | 31 (8.3) | | 12 (11.3) | | 43 (8.9) | |  | | 13 (9.8) | | 2 (6.4) | | 15 (9.1) | |  | |
| Agriculture | | 115 (30.8) | | 33 (31.1) | | 148 (30.9) | |  | | 38 (28.6) | | 11 (35.5) | | 49 (29.9) | |  | |
| Government | | 28 (7.5) | | 7 (6.6) | | 35 (7.3) | |  | | 11 (8.3) | | 2 (6.4) | | 13 (7.9) | |  | |
| Business | | 78 (20.9) | | 16 (15.1) | | 94 (19.6) | |  | | 28 (21.0) | | 8 (25.8) | | 36 (21.9) | |  | |
| Laborer | | 87 (23.3) | | 23 (21.7) | | 110 (22.9) | |  | | 31 (23.3) | | 6 (19.3) | | 37 (22.6) | |  | |
| Other | | 34 (9.1) | | 15 (14.2) | | 49 (10.2) | |  | | 12 (9.0) | | 2 (6.4) | | 14 (8.5) | |  | |
| **Intravenous drug user** | |  | |  | |  | | 0.004* | |  | |  | |  | | 0.541 | |
| No | | 316 (84.7) | | 76 (71.7) | | 392 (81.8) | |  | | 118 (88.7) | | 26 (83.9) | | 144 (87.8) | |  | |
| Yes | | 57 (15.3) | | 30 (28.3) | | 87 (18.2) | |  | | 15 (11.3) | | 5 (16.1) | | 20 (12.2) | |  | |
| **Recreational drug user** | |  | |  | |  | | 0.618 | |  | |  | |  | | 0.184 | |
| No | | 328 (87.9) | | 91 (85.8) | | 419 (87.5) | |  | | 113 (84.9) | | 23 (74.2) | | 136 (82.9) | |  | |
| Yes | | 45 (12.1) | | 15 (14.2) | | 60 (12.5) | |  | | 20 (15.1) | | 8 (25.8) | | 28 (17.1) | |  | |
| **Travel abroad** | |  | |  | |  | | 0.627 | |  | |  | |  | | 0.207 | |
| No | | 321 (86.1) | | 94 (88.7) | | 415 (86.6) | |  | | 120 (90.2) | | 25 (80.6) | | 145 (88.4) | |  | |
| Yes | | 52 (13.9) | | 12 (11.3) | | 64 (13.4) | |  | | 13 (9.8) | | 6 (19.4) | | 19 (11.6) | |  | |
| **Stilt house** | |  | |  | |  | | 0.088 | |  | |  | |  | | 0.658 | |
| No | | 313 (83.9) | | 78 (73.6) | | 391 (81.6) | |  | | 99 (74.4) | | 22 (71.0) | | 121 (73.8) | |  | |
| Yes | | 60 (16.1) | | 28 (26.4) | | 88 (18.4) | |  | | 34 (25.6) | | 9 (29.0) | | 43 (26.2) | |  | |
| **Raised animal** | |  | |  | |  | | 0.794 | |  | |  | |  | | 0.802 | |
| No | | 285 (76.4) | | 83 (78.3) | | 368 (76.8) | |  | | 109 (81.9) | | 25 (80.6) | | 134 (81.7) | |  | |
| Yes | | 88 (23.6) | | 23 (21.7) | | 111 (23.2) | |  | | 24 (18.1) | | 6 (19.4) | | 30 (18.3) | |  | |
| **Bed net** | |  | |  | |  | | 0.140 | |  | |  | |  | | 0.548 | |
| Yes | | 132 (35.4) | | 46 (43.4) | | 178 (37.2) | |  | | 56 (42.1) | | 11 (35.5) | | 67 (40.8) | |  | |
| No | | 241 (64.6) | | 60 (56.6) | | 301 (62.8) | |  | | 77 (57.9) | | 20 (64.5) | | 97 (59.2) | |  | |
| **Opportunistic infection** | |  | |  | |  | | 0.626 | |  | |  | |  | | 0.768 | |
| No | | 354 (94.9) | | 99 (93.4) | | 453 (94.6) | |  | | 115 (86.5) | | 28 (90.3) | | 143 (87.2) | |  | |
| Yes | | 19 (5.1) | | 9 (6.6) | | 26 (5.4) | |  | | 18 (13.5) | | 3 (9.7) | | 21 (12.8) | |  | |
| **Current Viral load** | |  | |  | |  | | 0.579 | |  | |  | |  | | 0.139 | |
| Undetectable | | 338 (90.6) | | 94 (88.7) | | 432 (90.2) | |  | | 118 (88.7) | | 24 (77.4) | | 142 (86.6) | |  | |
| Detectable | | 35 (9.4) | | 12 (11.3) | | 47 (9.8) | |  | | 15 (11.3) | | 7 (22.6) | | 22 (13.4) | |  | |
| **Current CD4 cell count** | |  | |  | |  | | 0.256 | |  | |  | |  | | 0.133 | |
| >500 cell/mm^3^ | | 275 (73.7) | | 71 (66.9) | | 346 (72.3) | |  | | 82 (61.6) | | 13 (41.9) | | 95 (57.9) | |  | |
| 201-500 cell/mm^3^ | | 65 (17.4) | | 26 (24.5) | | 91 (19.0) | |  | | 36 (27.1) | | 13 (41.9) | | 49 (29.9) | |  | |
| <=200 cell/mm^3^ | | 33 (8.8) | | 9 (8.5) | | 42 (8.8) | |  | | 15 (11.3) | | 5 (16.1) | | 20 (12.2) | |  | |

*P < 0.05 = statistically significance using the chi-square test
