# Supplementary material for: Incidence and persistence of asymptomatic Leishmania infection among HIV-infected patients in Trang province, Southern Thailand: A cohort study
Source: PLoS Negl Trop Dis. 2024 Oct 8;18(10):e0012581. doi: 10.1371/journal.pntd.0012581 (PMC11488742; doi:10.1371/journal.pntd.0012581)
Supplement: Attachment — Submitted filename: Response to reviewers final version.docx [file pntd.0012581.s003.docx]

**Response to reviewers:**

**Incidence and persistence of asymptomatic *Leishmania* infection among HIV-infected patients in Trang province, southern Thailand: a cohort study**

**Reviewer #1**

**Methods**

This study aimed to investigate the incidence and persistence of *Leishmania* infection among HIV-infected individuals in an affected area, Trang Province, Southern Thailand. The study also identified associated risk factors. We think the objectives proposed by the authors are perfectly articulated with the hypothesis or question that would motivate an expert on the subject. Inclusion criteria were established. It is a cohort longitudinal study that enrolled 386 participants in the HIV clinic, Trang Hospital, who initially tested negative for *Leishmania* infection during 2015-2016, and 133 individuals initially tested positive for *Leishmania* infection. Thus, follow-up visits of 519 participants occurred during 2018-2019. Direct Agglutination Test (DAT) and PCR identified incident and persistent *Leishmania* infection cases. It was calculated density and persistence of *Leishmania* infection.
Cox proportional-hazards regression analyses were performed to assess risk factors for the incidence of *Leishmania* infection. The proportional hazards assumption for Cox regression analysis was assessed using scaled Schoenfeld residuals. Hazard ratios (HRs) and 95% confidence intervals (CIs) were calculated for the associations, and P- values of 0.05 or less were considered statistically significant. All statistical analyses were conducted using STATA, version SE14.
- We think the objectives are clearly articulated with a clear testable hypothesis.
- We think the study design is appropriate to address the stated objectives.
- We think the population is clearly described and appropriated for the hypothesis tested.
- We think the sample size is adequate to ensure adequate power to address the hypothesis tested.
- We think the statistical analysis used supports the conclusions, too.
- There are not concerns about ethical or regulatory requirements.

About ethical or regulatory requirements: this study followed the ethical principles outlined in the Declaration of Helsinki. Ethical approval was obtained from the Institutional Review Board of the Royal Thai Army Medical Department (approval number: S034h/60). The written informed consent was obtained from all study participants before their inclusion in the study. Confidentiality and anonymity of the participants were ensured by assigning unique identification codes to the collected data.

**Results**

Regarding the results, the study enrolled 386 participants in the HIV clinic, Trang Hospital, who initially tested negative for *Leishmania* infection during 2015-2016, and 133 individuals initially tested positive for *Leishmania* infection. Among the initially negative group, incident cases comprised one L. orientalis infection and 11 seropositive cases using DAT, resulting in a cumulative incidence of 3.1% and an incidence density of 10.38 per 1000 person- years. Five persistent cases comprised one L. donovani complex and four seropositive cases using DAT in the initially positive group, with a cumulative persistence rate of 3.7% and a persistence density of 12.85 per 1000 person-years

-Does the analysis presented match the analysis plan? We think yes.
-Are the results clearly and completely presented? We think yes, too
-Are the figures (Tables, Images) of sufficient quality for clarity? Yes

**Conclusions**

This study sheds light on the incidence and persistence of *Leishmania* infection among HIV-infected individuals in Trang Province, Southern Thailand. It has immediate implications for healthcare providers, policymakers, and public health officials, aiding them in implementing targeted interventions and preventive measures to reduce the impact of leishmaniasis among HIV-infected individuals.

- Are the conclusions supported by the data presented? Yes.
- Are the limitations of analysis clearly described? Yes
- Do the authors discuss how these data can be helpful to advance our understanding of the topic under study? Yes.
- Is public health relevance addressed. Yes.

**Editorial and Data Presentation Modifications?**

WE COMMENT AND SUGGEST:
**Question #1.** In all the text there is a lack of standardization of the nomenclature of *Leishmania*.

**Answer:** We reviesed the nomenclature of *Leishmania* throughout the manuscript.

**Question #2.** Page 18, line 315. Among the positive cases, 11 individuals tested positive for *Leishmania* antibodies using DAT alone. DAT appears to the best serological test for leishmaniasis both in terms of sensitivity and specificity. But this reaction can cross with other diseases and this must be taken into consideration, especially considering immunosuppressed patients, therefore more vulnerable to infections. Furthermore, it is known that there are cases of Trypanosoma infection in Thailand. On the other hand, it is known that there is a window of false positivity or negativity in both tests used, DAT and PCR. Therefore, I think it would be appropriate to comment on these situations. To give more credibility to the results, it would be good to clarify whether there was more than one technician who read the DAT.

**Answer:** Lines 231-232 is revised as: "The DAT results were interpreted by two experienced technicians."

**Answer:** Lines 391-398 were added in the discussion section.

**Question #3.** Page. 20, line 342. Table 3. Patient number 5. There should be an effort by the authors to identify the *Leishmania* species in both the baseline diagnosis and follow-up to prove that the persistence of the infection was caused by the same species, reducing the possibility of a new infection.

**Answer:** We were concerned about species identification. Unfortunately, we faced the problem of DNA inadequate.

**Summary and General Comments**

- The claims are properly placed in the context of the previous literature.
- The authors have treated the literature fairly.
- The data and analyses fully support the claims.
- The results open perspectives for new researches as a multicentric in Thailand related to HIV infection patients.
- This is a paper outstanding in coinfection HIV/Leishmaniasis that inspires new questions about.
- The details of the methodology are sufficient to allow the experiments to be reproduced. They followed the checklist of items of STROBE guidelines for reporting observational studies.
- The manuscript is well organized and written clearly enough to be accessible to non-specialists.

**Do you want your identity to be public for this peer review?**

Yes: Raimunda Nonata Ribeiro Sampaio

**Reviewer #2**

**Methods**

Let me start by saying that I like the clear definitions provided in the methods on asymptomatic *Leishmania* infection, symptomatic VL, and seropositivity. The methods are described well enough, but are not entirely clear at some points. The objectives are mostly clear.

The thing I mostly have doubts on are the sample size, but this may be due to a misunderstanding in the methods (see minor comments below) on my part. While the total sample size seems large (n=643 baseline visit, n=519 follow-up), it seems that the authors stratify these groups to identify risk factors associated with incident *Leishmania* infection (n = 12). Am I correct in my understanding that the authors are comparing within the incident *Leishmania* group (n = 12) whether those with an age above 40 years old have a higher/lower hazard ratio than those below 40 years old? Thus, while the total n > 500, the n = 12 is for this analysis? It seems strange to me to compare within this group, rather than compare between the population-at-risk that did not have the event (so the n=374 'Negative' group) and those that did have the event (the n=12 'Incident *Leishmania* infection' cases).

**Answer:** For data analysis, we compared the population-at-risk that did not have the event (n=373* 'Negative' group) and those that did not (n=12 'Incidence of *Leishmania* infection' cases). In addition, we revised the title in Table 4 to "Table 4. Incident *Leishmania* infection, bivariate and multivariate Cox proportional-hazards regression analyses of risk factors for incident *Leishmania* infection (n=373, negative *Leishmania* infection at baseline; n = 12, *Leishmania* infection at follow-up)".

* We corrected the number of the Negative group to 373, as the previous report incorrectly stated it as 374.

In addition to this more major concern, there are a few things regarding the study design that are unclear and would benefit from some suggestions/comments to improve the clarity:

**Question #1.** In Line 102, where part of the objectives are already mentioned, it would be best to clearly define what the risk factors are for. It seems to be for incident *Leishmania* infection, rather than VL development. This needs to be made clear at this sentence already.

**Answer:** We changed the sentence from "Therefore, a critical need for cohort studies to address this knowledge gap is essential to provide insights into *Leishmania* infection incidence among HIV-infected patients and identify risk factors, including characteristics of persistent infection." to

In lines 122-124, "Therefore, a critical need for cohort studies to address this knowledge gap is essential to provide insights into *Leishmania* infection incidence with risk factors among HIV-infected patients, including characteristics of persistent infection."

**Question #2**. In the small paragraph where the authors define seropositivity, seroconversion can be defined also.

**Answer:** In lines 181-185 were revised as suggested.

**Question #3**. Relating to comment #2, the authors could benefit from mentioning how the studied population relates to the definitions provided, already in the methods, instead of the results. For example, the authors mention in the results on L268 that individuals testing negative for *Leishmania* at the baseline visit are the population at risk for incident *Leishmania* infection. This can be related to the definitions in the methods section already.

**Answer:** The information in lines 268-272 was moved to lines 181-185 in the method section.

**Question #4**. I believe it would be helpful if the authors would adjust Figure 1 to highlight what exactly they are comparing and to what objectives it relates.

**Answer:** Figure 1. was revised using highlighted color regarding the reviewer's comments.

**Question #5**. In the results section in Table 4, "Crude *Leishmania* infection" is mentioned. This is not mentioned anywhere in the methods section. What is this definition?

**Answer:** We revised the manuscript using the incidence density throughout the study and replaced Crude *Leishmania* infection with incidence density in Table 4. Incidence density was defined in lines 253-258.

**Question #6**. In this study, the 'asymptomatic *Leishmania* infection' is defined as testing positive for a DAT or nPCR. While I do not necessarily disagree on whether these can be defined as asymptomatic or not, they do omit VL history in this definition. Do the authors have any idea on the clinical history of the participants? Are those with seropositivity past VL cases, or are they truly 'resistant' asymptomatic cases? If the authors have this data, it would be wise to report it.

**Answer:** To clarify the definition of asymptomatic *Leishmania* infection, lines 164-166 were revised to be" Asymptomatic *Leishmania* infection, within the scope of this study, pertains to individuals who had no history of VL infection and did not exhibit any clinical symptoms associated with VL during the study period.".

**Question #7**. The statistics for the baseline characteristics in Table 1 were not reported. What statistical test was used?

**Answer:** We revised the information as suggested:

- Lines 252-253: "Descriptive statistics were used to summarize the baseline characteristics of the study population using the chi-square test.".
- Footnote S1 table *P < 0.05 = statistically significant using the chi-square test.

**Question #8**. Arbitrary categories for variables were made to test for, such as age <= 40 or age >40. Why not analyse this as a continuous variable? If you did not find any statistical difference between age <=40 and >40, would you then adjust to age <=50 and >50 to find a statistical difference? This hypothetical scenario highlights why you should not analyse some continuous variables as a categorical variable unless there is biological reasoning for the threshold.

**Answer:** We reanalyzed the age group into the continuous variables as suggested in Table 1 and Table 4.

**Results**

The more major concern I have with the analysis is similar as mentioned above under the 'Methods' comments. It is unclear whether the bivariate/multivariate Cox proportional-hazards regression was performed on only the n=12 incident *Leishmania* cases, or on the full population-at-risk?
In addition to this, I have a few minor suggestions to improve the clarity of the results section:

**Question #1.** Table 1 currently presents results stratified by follow-up status. This makes the table hard to interpret, and is really only interesting to validate whether the results are representable. As a suggestion, I would move this table to a supplementary file, while presenting the participant characteristics for either all patients or for only those patients that were followed-up in the main results as Table 1.

**Answer:** We moved Table 1 to the supplementary file and revised it as suggested in question number 2.

**Question #2**. In Table 1, it seems that statistical tests were only performed comparing within-group based on follow-up status? So the authors only compared between a 'Yes' and 'No' follow-up status for negative *Leishmania* infection, and separately for positive *Leishmania* infection? If this is correct, it would be beneficial to adapt this to comparing negative *Leishmania* infection to positive *Leishmania* infection, either at baseline or only for those followed-up.

**Answer:** We revised the table 1 as suggested.

**Question #3**. In Table 1, please define the units of the variables. For example, is age represented as n (%)?

**Answer:** we added n (%) in all categories.

**Question #4**. In Table 4, please define Crude *Leishmania* infection.

**Answer:** We revised the manuscript using the incidence density throughout the study and replaced Crude *Leishmania* infection with incidence density in Table 4. Incidence density was defined in lines 255-260.

**Question #5**. Am I correct in my understanding that there were no statistical tests for Occupation, Intravenous drug user, and Recreational drug user, in Table 4?

**Answer:** Yes, we did not include these variables: occupation, intravenous drug user, and recreational drug user in the Cox proportional-hazards regression analyses due to zero cases among the incident cases of intravenous drug users, recreational drug users, and unemployed.

**Conclusions**

With regard to public health relevance, this is clearly addressed. The authors can, however, be a bit more specific on how this data can be used to advance public health measures, with specific measures linked to the outcomes.

However, the conclusions are not always supported by the data presented. This relates to my major concern above.

**Answer:** We revised the conclusion as per suggestion in lines 518-531.

**Editorial and Data Presentation Modifications?**

Minor Revision

**Summary and General Comments**

The study by Bualert et al. focuses on the incidence of asymptomatic *Leishmania* infection in people living with HIV in a province of southern Thailand, and assesses several risk factors for *Leishmania* infection. This study in the most neglected subset (HIV-positive individuals) of already neglected patients is of crucial importance. One of the key strengths is the longitudinal design, with a baseline visit and a follow-up visit around 2-3 years later. I also like that they provide clear explanations on their definitions of asymptomatic *Leishmania* infection and symptomatic VL. However, the study results are not presented clearly at times, and can perhaps be clarified (see feedback/comments above and below).

**Major comments:**
**Question.** The thing I mostly have doubts on are the sample size, but this may be due to a misunderstanding in the methods (see minor comments below) on my part. While the total sample size seems large (n=643 baseline visit, n=519 follow-up), it seems that the authors stratify these groups to identify risk factors associated with incident *Leishmania* infection (n = 12). Am I correct in my understanding that the authors are comparing within the incident *Leishmania* group (n = 12) whether those with an age above 40 years old have a higher/lower hazard ratio than those below 40 years old? Thus, while the total n > 500, the n = 12 is for this analysis? It seems strange to me to compare within this group, rather than compare between the population-at-risk that did not have the event (so the n=374 'Negative' group) and those that did have the event (the n=12 'Incident *Leishmania* infection' cases). If this is truly the case, I would suggest to re-do this analysis.

**Answer:** The reviewer is correct about the analysis of incidence density. We compared the population-at-risk that did not have the event (n=373) to those that did have the event (n=12).

To clarify, we did not perform the analysis within the incident *Leishmania* group alone. Instead, we compared the two groups: those who remained negative for *Leishmania* infection (n=373) and those who developed incident *Leishmania* infection (n=12). This approach allows us to identify risk factors associated with the incidence of *Leishmania* infection more accurately.

To calculate the incidence density, we divided the number of new cases by the total follow-up time contributed by each group. This methodology is reflected in Table 4. Additionally, per your suggestion, we reanalyzed the age variable, treating it as a continuous variable rather than a categorical one, to ensure a more robust statistical analysis.

* We corrected the number of the Negative group to 373, as the previous report incorrectly stated it as 374.

**Minor comments:**

**Question #1.** In the Author Summary, on L68-L69, the authors state that "Even individuals who initially tested negative for *Leishmania* are not immune to infection'. Is this not already obvious? This study does not reveal this, and no researcher thinks that is the case. Consider to rewrite or remove this sentence.

**Answer:** We revised lines 69-70 to be (Firstly, this study revealed a significant incidence rate of 3.2% among HIV patients, highlighting the ongoing risk of *Leishmania* transmission in the study area.).

**Question #2.** In line with comment #1, the authors write on L363-L367 that individuals with an initial negative status are considered to be at a lower risk of *Leishmania* infection. This is definitely not considered. A negative infection test does not mean an individual is at a lower risk of future infection; it only states that a person is not infected at that time.

**Answer:** We deleted "even among those considered at lower risk due to their initial negative status." from the sentence. (Lines 404-407)

**Question #3.** It is not clear what is meant in L40-L42 with 'reveals little information'.

**Answer:** We revised lines 40-42: "Thailand, *Leishmania* infection caused by *Leishmania matiniquensis* and *Leishmania orientalis* lacks information about the incidence and risk factors among HIV-infected populations."

**Question #4.** On L44-L45, please define what the risk factors are associated with. In this case, it seems to be incident Leishmania infection, so the risk of contracting a *Leishmania* infection. This is important to define, as risk factors for infection can be different from disease development.

**Answer:** We revised lines 44-45: "The study also identified associated risk factors of incidence of *Leishmania* infection."

**Question #5.** On L80-81, the authors mention that leishmaniasis is prevalent globally in the Indian subcontinent and East Africa. What about South-America, or Asia, such as Thailand where the study situates?

**Answer:** We revised lines 80-81, "Leishmaniasis is prevalent globally in the Indian subcontinent, East Africa, and South America."

In lines 93-95, "In southeast Asia, especially Thailand, leishmaniasis, mainly caused by *Leishmania martiniquensis* and *Leishmania* *orientalis*, has been reported in various regions, including Trang Province, southern Thailand."

**Question #6.** The introduction does not touch upon any already known risk factors for incident *Leishmania* infection, even on other continents and in other regions. Consider to introduce this already.

**Answer:** In the introduction, the information on known risk factors for the incidence of *Leishmania* infection is added in lines 108-118.

**Question #7.** On Line 102, where part of the objectives are already mentioned, it would be best to clearly define what the risk factors are for. It seems to be for incident *Leishmania* infection, rather than VL development. This needs to be made clear at this sentence already.

**Answer:** We already answered in the above section.

**Question #8.** In the small paragraph where the authors define seropositivity, seroconversion can also be defined.

**Answer:** We already answered in the above section.

**Question #9.** Relating to comment #2, the authors could benefit from mentioning how the studied population relates to the definitions provided, already in the methods, instead of the results. For example, the authors mention in the results on L268 that individuals testing negative for *Leishmania* at the baseline visit are the population at risk for incident *Leishmania* infection. This can be related to the definitions in the methods section already.

**Answer:** We already answered in the above section.

**Question #10.** I believe it would be helpful if the authors would adjust Figure 1 to highlight what exactly they are comparing and to what objectives it relates.

**Answer:** We already answered in the above section.

**Question #11.** In the results section in Table 4, "Crude *Leishmania* infection" is mentioned. This is not mentioned anywhere in the methods section. What is this definition?

**Answer:** We already answered in the above section.

**Question #12.** In this study, the 'asymptomatic *Leishmania* infection' is defined as testing positive for a DAT or nPCR. While I do not necessarily disagree on whether these can be defined as asymptomatic or not, they do omit VL history in this definition. Do the authors have any idea on the clinical history of the participants? Are those with seropositivity past VL cases, or are they truly 'resistant' asymptomatic cases? If the authors have this data, it would be wise to report it.

**Answer:** We already answered in the above section.

**Question #13.** On L162-L163, the authors mention that the individuals continued to harbor an active and detectable *Leishmania* infection through-out the follow-up period. Did they consistently do periodic testing from the baseline visit until the follow-up visit, or do they mean that participants that tested positive on the baseline visit still tested positive years later on the follow-up visit? This is an important difference.

**Answer:** We did not do periodic testing from the baseline visit until the follow-up visit. However, all participants were clinically observed every six months.

Lines 186-189: "The persistence of *Leishmania* infection pertains to cases of the disease among HIV-infected patients who initially tested positive for leishmaniasis during the baseline enrollment between 2015 and 2016 and detectable *Leishmania* infection at the follow-up period from 2018 to 2019."

**Question #14.** In the methods section, on L163-L165, the authors write about their defined persistent cases not achieving complete cure. However, there is no mention of treatment, nor of past VL history. Were these participants ever treated? Or could it be a re-infection rather than persistence? This is not adequately discussed and there is too little information provided to speculate.

**Answer:** Lines 186-189 were revised. There was no treatment of *Leishmania* infection in this study population since all of them were asymptomatic cases. However, they have been closely observed every six months for any clinical related to *Leishmania* infection at the HIV clinic. As suggested, we also added the concern about the reinfection in the discussion (lines 451-457).

**Question #15.** The statistics for the baseline characteristics in Table 1 were not reported. What statistical test was used?

**Answer:** We already answered in the above section.

**Question #16.** In Table 1, it seems that statistical tests were only performed comparing within-group based on follow-up status? So the authors only compared between a 'Yes' and 'No' follow-up status for negative *Leishmania* infection, and separately for positive *Leishmania* infection? If this is correct, it would be beneficial to adapt this to comparing negative *Leishmania* infection to positive *Leishmania* infection, either at baseline or only for those followed-up.

**Answer:** We already answered in the above section.

**Question #17.** Table 1 currently presents results stratified by follow-up status. This makes the table hard to interpret, and is really only interesting to validate whether the results are representable. As a suggestion, I would move this table to a supplementary file, while presenting the participant characteristics for either all patients or for only those patients that were followed-up in the main results as Table 1.

**Answer:** We already answered in the above section.

**Question #18.** In Table 1, please define the units of the variables. For example, is age represented as n (%)?

**Answer:** We already answered in the above section.

**Question #19.** The paragraph' Investigation of persistent *Leishmania* infection' on L285 seems to be about the positive *Leishmania* infection group, and not the persistent group. Reconsider the title.

**Answer:** Lines 309-310 were revised as "The population at Risk for Persistent *Leishmania* Infection (Tested Positive at Baseline, n=164)"

**Question #20.** In Table 4, please define Crude *Leishmania* infection.

**Answer:** We already answered in the above section.

**Question #21.**. Am I correct in my understanding that there were no statistical tests for Occupation, Intravenous drug user, and Recreational drug user, in Table 4?

**Answer:** We already answered in the above section.

**Question #22.** In the discussion, the authors compare incidence rates of this study to those in other settings such as North-West Ethiopia. However, these are different incidence rates. This present study defines the incidence of *Leishmania* infection, while those study assess leishmaniasis cases (so disease incidence). Consider to mention this discrepancy.

**Answer:** Lines 409-421 were revised in the discussion section.

**Question #23.** Arbitrary categories for variables were made to test for, such as age <= 40 or age >40. Why not analyse this as a continuous variable? If you did not find any statistical difference between age <=40 and >40, would you then adjust to age <=50 and >50 to find a statistical difference? This hypothetical scenario highlights why you should not analyse some continuous variables as a categorical variable unless there is biological reasoning for the threshold.

**Answer:** We already answered in the above section.

**Question #24.** Relating to comment #23, since you identify age as a significant associated factor with a categorical arbitrary split, consider to include an analysis where you use it as a continuous variable.

**Answer:** We already answered in the above section.

**Question #25.** There is no reference provided for L445-L446 where the authors state that individuals with lower CD4 counts are at a higher risk of *Leishmania* infection. Most of the literature on CD4 counts states it is a risk factor particularly for VL development (and subsequent relapse), and not particularly for infection. This is an important distinction that the authors sometimes do not make.

Answer: References were added on lines 501- 504.

**Do you want your identity to be public for this peer review?**

Yes: Nicky de Vrij

**Reviewer #3**

**Methods**

-Are the objectives of the study clearly articulated with a clear testable hypothesis stated?
Answer: Yes
-Is the study design appropriate to address the stated objectives?
Answer: Yes
-Is the population clearly described and appropriate for the hypothesis being tested?
Answer: Yes
-Is the sample size sufficient to ensure adequate power to address the hypothesis being tested?
Answer: Yes
-Were correct statistical analysis used to support conclusions?
Answer: Yes
-Are there concerns about ethical or regulatory requirements being met?
Answer: No

**Results**

-Does the analysis presented match the analysis plan?
Answer: yes
-Are the results clearly and completely presented?
Answer: yes
-Are the figures (Tables, Images) of sufficient quality for clarity?
Answer: no

**Conclusions**

-Are the conclusions supported by the data presented?
Answer: no
-Are the limitations of analysis clearly described?
Answer: no
-Do the authors discuss how these data can be helpful to advance our understanding of the topic under study?
Answer: yes
-Is public health relevance addressed
Answer: yes

**Editorial and Data Presentation Modifications?**

(No Response)

**Summary and General Comments**

In this manuscript, the authors evaluated the incidence and persistence of asymptomatic *Leishmania* infection in people living with HIV in the southern region of Thailand. The topic is very interesting and relevant for understanding *Leishmania* and HIV co-infection. The manuscript has very relevant information, however some points need to be made clearer to make the work more robust. Below, I send my review and suggestions.
**Introduction:**
**Question #1.** I suggest include a paragraph regarding the sensitivity and sensibility of the methods used to detect *Leishmania* infection.

**Answer:** DAT and nPCR sensitivity were added in introduction lines 98-107.

**Question #2.** I suggest include new lasted others information about coinfection *Leishmania* and HIV, mainly those used different methodology to detect *Leishmania* infection.
**Answer:** We revised the manuscript as suggested in lines 464-472.

**Methodology**
**Question #1.** I suggest include any other methodology to detect antibodies, not only DAT.

Answer: We revised the manuscript as suggested in lines 98-107.

**Question #2.** Regarding DAT, what the *Leishmania* specie and strain used as antigens? It is an important point, due to reaction with the *Leishmania* species.
**Answer:** Lines 225-226 were added. "The DAT contained freeze-dried *Leishmania* antigen was prepared using *Leishmania donovani* promastigotes."

**Results**
**Question #1.** In the table 1, I suggest counting CD4 lymphocytes at three levels (<200, >200 and <500, >500) to assess whether there was a difference in incidence and persistence of infection.

**Answer:** We revised the category of CD4 lymphocytes at three levels per suggestion in tables 1 and 4 (>500 cell/mm^3^, 201-500 cell/mm^3^ and <=200 cell/mm^3^)

**Question #2.** Regarding the identification of infection by *L. (V.) lainsoni*, it would be essential to clarify the epdemiology of this patient, as this species, as well as the others in the subgenus Viannia, only occur in Latin America. It would be essential to review this result, as there may have been an error in identifying the species or the patient may have become infected when traveling to an area where *L. lainsoni* occurs.

Answer: We revised in the discussion, lines 458-463.

**Discussion:**
An important point to take into consideration in the discussion is the methodology used to determine infection. It is known that the methodologies have different sensitivity. Another point to be taken into consideration in the discussion refers to the specificity of the DAT. There is a description in the literature about cross-reactivity of serological methods for diagnosing leishmaniasis with other diseases, such as tuberculosis. Cunha et al obtained different frequency percentages, using different methodologies to detect *Leishmania* infection in people living with HIV.(Cunha MA, Celeste BJ, Kesper N et al. Frequency of *Leishmania* spp. infection among HIV-infected patients living in an urban area in Brazil: a cross-sectional study. BMC Infect Dis. 2020 Nov 25;20(1):885). I suggest consider this information in the discussion.
**Answer:** We revised in the discussion, lines 458-463.

**Do you want your identity to be public for this peer review?**

No
