# Supplementary material for: Incidence and persistence of asymptomatic Leishmania infection among HIV-infected patients in Trang province, Southern Thailand: A cohort study
Source: PLoS Negl Trop Dis. 2024 Oct 8;18(10):e0012581. doi: 10.1371/journal.pntd.0012581 (PMC11488742; doi:10.1371/journal.pntd.0012581)
Supplement: Attachment — Submitted filename: Response to reviewers version2.docx [file pntd.0012581.s004.docx]

**Response to reviewers:**

**Incidence and persistence of asymptomatic *Leishmania* infection among HIV-infected patients**

**Reviewer 3**

**Summary and General Comments**
**Question 1:** The way the results are presented is confusing. For example, regarding Leishmania infection in the baseline, it is not clear whether the 164 positive cases were due to antibody detection or Leishmania DNA detection. I suggest making this information clearer. I think it should show how many are serologically positive and how many of these had a positive PCR test, initially.

Answer: We revised the information in lines 275-288 and figure 1.

Figure 1 presents an overview of the study's baseline data from 2016, detailing the enrollment of 643 HIV-infected individuals, their Leishmania infection test results, and the subsequent follow-up outcomes in 2019. At baseline, 164 individuals tested positive for Leishmania infection, with 109 being DAT positive only, 44 nPCR positive only, and 11 positives by both DAT and nPCR, while 479 individuals tested negative. During the follow-up period in 2019, 373 of the 479 individuals who were negative at baseline (77.9%) were followed up, with 12 (3.2%) testing positive for Leishmania infection, indicating incident cases. Among the 164 individuals who were positive at baseline, 133 (81.1%) were followed up; of these, five individuals (3.7%) remained positive, indicating persistent infection, with 91 initially DAT positive only, 35 nPCR positive only, and 7 positives by both DAT and nPCR. Meanwhile, 128 individuals (96.3%) who were positive at baseline tested negative during follow-up, indicating clearance of the infection. Loss to follow-up was noted in 106 (22.1%) of the individuals who were negative at baseline and 31 (18.9%) of those who were positive at baseline. The median follow-up time was 2.9 years (IQR: 2.5-3.4 years).

**Question 2:** Regarding the persistence of the infection, what is the methodology, DAT only? How many DAT positives were tested by PCR and how many were positive by PCR?

**Answer:** We revised the information in lines 189, 349-359

The persistence of Leishmania infection pertains to cases of the disease among HIV-infected patients who initially tested positive for leishmaniasis during the baseline enrollment between 2015 and 2016 and detectable Leishmania infection during the follow-up period from 2018 to 2019 where positivity was determined using nPCR and DAT.

The persistence of Leishmania infection pertains to cases among HIV-infected patients who initially tested positive for leishmaniasis during the baseline enrollment between 2015 and 2016, where positivity was determined using the Direct Agglutination Test (DAT) and nPCR. At follow-up between 2018 and 2019, among the 133 individuals who were followed up after initially testing positive, five individuals (3.7%) remained positive, indicating persistent infection. Regarding the methodology used to assess persistence, both DAT and nPCR were utilized during the follow-up period. Specifically, all 133 individuals who tested positive at baseline were retested using DAT and nPCR during follow-up.

Table 2 shows the results of DAT and species identification among 133 Leishmania infections at the baseline visit (2015-2016). During the follow-up period (2018-2019), five cases remained identified as testing positive for Leishmania infections (Table 3).

**Question 3:** Regarding Table 2. These results are completely confusing. I don't understand. I suggest you review this information. Apparently, of the 133 patients with positive DAT in the Baseline, only 7 had Leishmania DNA detected. In 98, DAT was performed and 98 had positive DAT. In another 35, only sequencing was performed and no PCR was performed?? According to what is shown in the table, 7 patients showed positive DAT and positive nPCR, is that it?

**Answer:** The table 2 was revised.

| Diagnosis of Leishmania infection at Baseline visit (2015-2016) and enrolled at the follow-up visit (2018-2019) n = 133 | | | | |
| --- | --- | --- | --- | --- |
|  | Positive by DAT only (n=91) | Positive by nPCR  (n =35) | Positive by DAT and nPCR  (n =7) | Total (n=133) |
| DAT 1:100 | 6 |  |  |  |
| DAT 1:200 | 12 |  |  |  |
| DAT 1:400 | 38 |  | *L. martiniquensis* (1),  *Leishmania.* Spp. (2) |  |
| DAT 1:800 | 26 |  | *L. orientalis* (3) |  |
| DAT 1:1600 | 9 |  | *L. orientalis* (1) |  |
| **Species identification** |  |  |  |  |
| *L. orientalis* |  | 13 |  |  |
| *L. donovani complex* |  | 7 |  |  |
| *L. martiniquensis* |  | 5 |  |  |
| *L. lainsoni* |  | 4 |  |  |
| *Leishmania* spp. |  | 6 |  |  |
|  | 91 | 35 | 7 | 133 |

**Question 4:** The authors continue to insist on the infection by L. lainsoni, however the results presented do not confirm the infection by L. lainsoni. I believe that this information is biased, since only L. lainsoni was detected using the ITS-1 target and even when using kDNA (9, which has more copies) the PCR was negative. This information is not real.
**Answer:** We revised the discussion in lines 465-469.
In this study*, L. lainsoni* infection was identified using the ITS1-PCR; this species was initially reported in Latin America (26). This patient had no history of traveling abroad. Unfortunately, the effort to confirm *L. lainsoni* infection using other target genes, i.e., *hsp70*-PCR and *kDNA* PCR, was unsuccessful and negative PCR results were obtained. Therefore *L. lainsoni* is still a doubtful species identification.
